# Surveillance During REM Sleep for the First-Night Effect

**DOI:** 10.3389/fnins.2019.01161

**Published:** 2019-10-29

**Authors:** Masako Tamaki, Yuka Sasaki

**Affiliations:** Department of Cognitive, Linguistic, and Psychological Sciences, Brown University, Providence, RI, United States

**Keywords:** first-night effect, REM sleep, evoked brain response, theta activity, delta activity, default-mode network

## Abstract

We experience disturbed sleep in a new place, and this effect is known as the first-night effect (FNE) in sleep research. We previously demonstrated that the FNE is associated with a surveillance system in one brain hemisphere during NREM sleep, which manifests as interhemispheric asymmetry in sleep depth in the default-mode network (DMN) and increased vigilance toward monitoring external stimuli. This surveillance system may be useful for protecting vulnerable sleepers from abnormal events in unfamiliar environments. The present study investigated whether a similar surveillance system is exhibited during rapid eye movement (REM) sleep. The impacts of the FNE could be different between the phasic period, in which eyes move rapidly, and the tonic period, in which eye movement ceases, of REM sleep; without the FNE, vigilance to external stimuli is generally reduced during the phasic period but not the tonic period. Thus, REM sleep was split into phasic and tonic periods. First, we replicated previous findings showing interhemispheric asymmetry in delta activity in the DMN associated with the FNE during NREM sleep. However, during REM sleep, interhemispheric asymmetry in delta activity or theta activities, two oscillatory activities during REM sleep, was not found during the phasic or tonic periods. Next, we tested whether vigilance, as measured by evoked brain responses (P2) to deviant tones, associated with the FNE was increased in one hemisphere during REM sleep. The P2 amplitudes during the phasic period were augmented by the FNE on day 1 and were significantly larger than those on day 2 when the FNE was not present. In contrast, the P2 amplitudes during the tonic period were not different across days. The P2 amplitudes showed no interhemispheric asymmetry during the phasic or tonic periods. These results suggest that while the surveillance system exhibits interhemispheric asymmetry in sleep depth and vigilance during NREM sleep, this system shows no interhemispheric asymmetry in oscillatory activities and exhibits increased vigilance in both hemispheres only during the phasic period of REM sleep. Therefore, the surveillance system associated with the FNE may involve different mechanisms during NREM and REM sleep.

## Introduction

Sleep is crucial for the maintenance of daily life (Stickgold, 2005; Imeri and Opp, 2009). The psychological and behavioral consequences of a decline in sleep quality may be severe (Carskadon and Dement, 1981; Drummond et al., 2000; Buysse et al., 2011; Okawa, 2011; Czeisler, 2013). However, sleep quality may be decreased as a protective mechanism under specific circumstances, for example, sleeping deeply in only one brain hemisphere when an environment is not safe for the animal to sleep deeply in both hemispheres (Rattenborg et al., 1999; Peever and Fuller, 2017). One form of this mechanism is the first-night effect (FNE), which is widely known in human sleep research (Agnew et al., 1966; Carskadon and Dement, 1979; Tamaki et al., 2005a, b, 2014, 2016). The FNE is a temporary sleep disturbance that occurs specifically in the first session of sleep experiments in young healthy adults, and it manifests in prolonged sleep-onset latency, frequent arousal, and decreased deep non-rapid eye movement (NREM) sleep (Roth et al., 2005). Our previous study demonstrated that the FNE was not merely a sleep disturbance but a manifestation of a surveillance system in one hemisphere that remains less asleep and more vigilant than the other hemisphere (Tamaki et al., 2016). This surveillance system in one vigilant brain hemisphere may be useful for monitoring unfamiliar surroundings to protect sleepers in their most vulnerable state and to detect deviant events (Tamaki et al., 2016). We found that slow-wave activity or delta activity, which is an index of sleep depth, decreases in the left hemisphere compared to the right hemisphere regionally in the default-mode network (DMN) during NREM stage 3 sleep (stage N3 or slow-wave sleep) as part of the FNE on day 1, and this decrease causes interhemispheric asymmetry in slow-wave activity. The amplitude of an evoked brain response during stage N3 correlates with vigilance, and the amplitude was increased in one hemisphere on day 1, which caused interhemispheric asymmetry in vigilance. These interhemispheric asymmetries in local sleep depth and vigilance in monitoring the external world function as a surveillance system to counteract vulnerability during deep NREM sleep (Tamaki et al., 2016).

In contrast to NREM sleep, whether the FNE influences brain activities during rapid eye movement (REM) sleep and its possible mechanisms are less well-understood. Approximately 30 studies investigated the FNE during REM sleep in healthy young adults. The characteristics of the FNE during REM sleep include decreased time spent in REM sleep (Rechtschaffen and Verdone, 1964; Kales et al., 1967b; Sforza et al., 2008), delayed onset of REM sleep (Agnew et al., 1966; Kales et al., 1967b), and increased microarousals during REM sleep (Sforza et al., 2008). To the best of our knowledge, only two papers (Toussaint et al., 1997; Curcio et al., 2004) have investigated brain activities quantitatively during REM sleep. However, the results from these studies are contradictory. One study showed that the FNE decreased electroencephalography (EEG) power in a wide range of frequency bands, including delta (0.5–3.5 Hz), theta (4–7.5 Hz), and beta (13–21.5 Hz) bands (Toussaint et al., 1997). The other study reported that the FNE increased theta power (5 Hz) (Curcio et al., 2004). No study has investigated brain activities separately for each hemisphere or whether the FNE altered vigilance during REM sleep. Therefore, whether the FNE alters sleep depth and vigilance levels during REM sleep, acting as a surveillance system in an analogous manner to asymmetries in NREM sleep, is not known.

Notably, it is suggested that the ability to process external stimuli differs depending on whether REMs appear (phasic period) or not (tonic period) during REM sleep (Price and Kremen, 1980; Sallinen et al., 1996; Takahara et al., 2002, 2006b; Wehrle et al., 2007). First, the amplitudes of evoked brain responses differ significantly between the phasic and tonic periods; that is, much smaller brain responses are observed during the phasic period than during the tonic period (Sallinen et al., 1996; Takahara et al., 2002, 2006b). The amplitudes of evoked potentials correlate with the degree of vigilance (Nielsen-Bohlman et al., 1991; Michida et al., 2005). Thus, finding smaller potentials during the phasic period suggests that the degree of vigilance to external stimuli is significantly lower when the eyes are moving rapidly during the phasic period compared to tonic periods during REM sleep without the FNE (Michida et al., 2005). Second, one study investigated blood-oxygen-level dependency (BOLD) responses to acoustic stimulations (Wehrle et al., 2007) and reported that acoustic stimulations during the tonic period induced BOLD activations in the auditory cortex to some degree. In contrast, BOLD activations in the auditory cortex during the phasic period were only minimal. Thus, the ability to process external stimuli may be reduced significantly during the phasic period but sustained to some extent during the tonic period. These reports suggest that it is important to split REM sleep into phasic and tonic periods for the examination of evoked brain responses.

The present study investigated whether the FNE affected oscillatory activity and vigilance during REM sleep and whether decreased oscillatory activity or enhanced vigilance, if any, shows interhemispheric asymmetry in an analogous manner to that seen during NREM sleep. Theta activity was investigated because it is one of the major spontaneous brain oscillations during REM sleep (Takahara et al., 2006a). We also investigated delta activity during REM sleep because previous studies have shown that delta activity also occurs during REM sleep (Simor et al., 2016; Bernardi et al., 2019). We targeted the DMN for the analysis of theta and delta activities during REM sleep because a previous study (Tamaki et al., 2016) suggested that the DMN may be involved in the surveillance system. The study suggested that brain activities may need to be source-localized using individual anatomical brain information for the detection of asymmetry in delta activity. Moreover, interhemispheric asymmetry in delta activity is difficult to identify on the sensor space, at least in the case of NREM sleep (Tamaki et al., 2016). Therefore, we tested whether the FNE involved interhemispheric asymmetry in oscillatory activity in the DMN during REM sleep using a source-localization technique that combines EEG, structural MRI, and polysomnography (PSG) in the sleeping brain. To test whether the FNE altered vigilance, we measured evoked brain responses to external stimuli using an oddball paradigm during tonic and phasic periods. An evoked brain potential during REM sleep, the latency of which is approximately 200 ms (hereafter, P2), is elicited to deviant tones during REM sleep (Bastuji et al., 1995; Sallinen et al., 1996; Takahara et al., 2002, 2006b). Previous studies have found that the amplitude of P2 reflects the degree of vigilance during REM sleep (Takahara et al., 2006b). Thus, we used the P2 amplitude to test whether vigilance was enhanced in one hemisphere in the FNE during REM sleep analogous to that seen during NREM sleep. We also tested whether the impact of the FNE on brain responses differed between the phasic and tonic periods because the FNE may differentially influence these periods.

## Materials and Methods

### Participants

Twenty-four subjects participated in the present study. Twelve subjects participated in Experiment 1 (nine females; 23.3 ± 0.99 years old, mean ± SEM), and the other 12 subjects participated in Experiment 2 (six females; 24.8 ± 0.67 years old, mean ± SEM). An experimenter thoroughly described the purpose and procedures of the experiment to the candidate subjects, and they were asked to complete questionnaires about their sleep-wake habits for screening, including usual sleep and wake times, regularity of their sleep-wake habits and lifestyle, nap-taking habits, and information on their physical and psychiatric health, including sleep complaints. The exclusion criteria included having physical or psychiatric disease, currently receiving medical treatment, having a suspected sleep disorder, and having a habit of consuming alcoholic beverages before sleep or smoking. Eligible people had regular sleep-wake cycles, i.e., the difference between average bedtimes and sleep durations on weekdays and weekends was less than 2 h, and the average sleep duration regularly ranged from 6 to 9 h. All subjects gave written informed consent for their participation in the experiments. Data collection was performed at Brown University. The institutional review board approved the research protocol.

Four subjects’ data from day 1 and another 2 subjects’ data from day 2 were excluded from Experiment 2, resulting in complete data sets from 8 participants on day 1 and 10 participants on day 2. The data omitted from day 1 and day 2 were from different subjects. Therefore, there were 6 subjects whose data were available for both day 1 and day 2 in Experiment 2. Data were omitted because of a lack of REM sleep (*n* = 5) or because the measured brain responses were too noisy (*n* = 1).

### Experimental Design

Subjects in Experiments 1 and 2 were instructed to maintain their regular sleep-wake schedule, i.e., their daily wake/sleep time and sleep duration, until the study was over. The sleep-wake schedule of the subjects were monitored using a sleep log for 3 days prior to the experiment. Subjects were instructed to refrain from alcohol consumption, unusual excessive physical exercise, and naps on the day before the sleep session. Caffeine consumption was not allowed on the day of the experiments.

Both Experiments 1 and 2 were conducted with a nap design in accordance with a previous study (Tamaki et al., 2016), which found interhemispheric asymmetry in delta activity during NREM sleep with increased vigilance in one hemisphere in association with the FNE during the first nap session. To fairly compare NREM sleep and REM sleep, we used a daytime nap design in the current study. The impact of the FNE does not seem to be significantly different between daytime nap and night sleep (Tamaki et al., 2016).

#### Experiment 1

We tested whether there was hemispheric asymmetry in delta or theta activity in the DMN during REM sleep with the FNE. In addition, we tested whether interhemispheric asymmetry in delta activity in the DMN could be replicated during NREM sleep. All the subjects took a nap for the first time in the sleep laboratory. Subjects came to the experimental room at approximately 1 pm for PSG preparations. Room lights were turned off at approximately 2 pm, and the 90-min sleep session began. The time for the sleep session was chosen due to the known “mid-afternoon dip,” which would facilitate the onset of sleep, even in subjects who do not customarily nap (Horikawa et al., 2013). Structural MRI was measured on another day after the sleep session (see section “Anatomical MRI Acquisition and Region of Interest”). Approximately 1 week later, the same subjects came to the experiment room again and participated in a learning experiment. After the learning experiment, at approximately 1 pm, PSG was prepared, and the subjects were asked to go to sleep at approximately 2 pm for 90 min. These sleep sessions were performed approximately 1 week apart so that any effects of napping during the first sleep session would not carry over into the second sleep session. We only used the sleep-onset latency from the PSG data from the second sleep session to confirm the FNE on day 1. Other data, including oscillatory activities and the sleep structure during the second sleep session, were not analyzed for the current study, as the learning experiment prior to the sleep session was likely to modulate the results.

#### Experiment 2

We measured the evoked brain responses in each hemisphere during REM sleep using an oddball paradigm. We tested whether the evoked brain responses to deviant tones were larger on day 1 than on day 2. Subjects participated in two experimental sleep sessions (day 1 and day 2). These sessions were performed approximately 1 week apart so that any effects of napping during the first sleep session would not carry over into the second sleep session.

Subjects came to the experimental room at approximately 1 pm on both days, and electrodes were attached for PSG measurements (see section “PSG Measurement”). Subjects were taken to a sleep chamber after the electrodes were attached. Subjects were informed that faint beeping sounds may be presented through earphones while they slept, but they were instructed to ignore these sounds. Room lights were turned off at approximately 2 pm, and the 90-min sleep session began, as in Experiment 1. PSG was monitored, and experienced experimenters scored sleep stages in real time during the sleep session. Sound presentations (see “Auditory Stimuli”) began after at least 5 min of uninterrupted NREM stage 2 sleep. Sounds were presented during NREM and REM sleep, but sound presentations were stopped every time a lighter stage of sleep (stage W or NREM stage 1 sleep) or a period of arousal were observed. This procedure was repeated throughout the sleep session. See Supplementary Figure 1 for the total number of sound presentations for the tonic and phasic periods for all the subjects on days 1 and 2 (see section “Classification of Phasic and Tonic Periods”).

### PSG Measurement

Polysomnography was recorded in a soundproof and shielded room. PSG consisted of EEG, electrooculogram (EOG), electromyogram (EMG), and electrocardiogram (ECG) measurements. EEG was recorded at 64 scalp sites, according to the 10% electrode position (Sharbrough et al., 1991), using active electrodes (actiCap, Brain Products, LLC) with a standard amplifier (BrainAmp Standard, Brain Products, LLC). The online reference was Fz, which was re-referenced to the average of the left and right mastoids offline after recording. The sampling frequency was 500 Hz. The impedance was kept below 20 kΩ because the active electrodes included a new type of integrated impedance converter that allowed the EEG signal to be transmitted with significantly lower levels of noise than traditional passive electrode systems. The data quality obtained using the active electrodes was as good as the 5 kΩ quality obtained using passive electrodes, which were used for EOG and EMG recordings (BrainAmp ExG, Brain Products, LLC). The horizontal EOG was recorded from two electrodes placed at the outer canthi of each eye. The vertical EOG was measured from two electrodes 3 cm above and below each eye. EMG was recorded from the mentum (chin). ECG was recorded from two electrodes placed at the right clavicle and the left rib bone. The impedance was kept below 10 kΩ for the passive electrodes. Brain Vision Recorder software (Brain Products, LLC) was used for recording. The data were filtered between 0.1 and 40 Hz.

### Sleep-Stage Scoring and Sleep Parameters

Sleep stages were scored for every 30-s epoch, following standard criteria (Rechtschaffen and Kales, 1968; Iber et al., 2007), into the stages of wakefulness (stage W), NREM stage 1 sleep (stage N1), NREM stage 2 sleep (stage N2), NREM stage 3 sleep (stage N3), and REM sleep. The following variables were calculated for each subject to assess basic sleep structure (Table 1): the duration (min) and percentage (%) of time in each sleep stage, latency to REM sleep (min), sleep onset latency (SOL, min), wake time after sleep onset (WASO, min), sleep efficiency (SE,%), and time in bed (TIB, min).

**TABLE 1:** Sleep parameters.

	**Experiment 1**	**Experiment 2**	
	**Day 1**	**Day 1**	**Day 2**
	**(Mean ± SEM)**	**(Mean ± SEM)**	**(Mean ± SEM)**
Stage W (min)	10.6 ± 1.91	10.8 ± 1.83	5.0 ± 0.85
Stage N1 (min)	5.8 ± 1.78	8.9 ± 2.02	4.9 ± 1.07
Stage N2 (min)	36.6 ± 3.98	34.5 ± 2.42	30.3 ± 3.15
Stage N3 (min)	12.7 ± 3.57	12.6 ± 2.12	19.5 ± 2.88
REM sleep (min)	14.8 ± 2.20	9.3 ± 1.83	15.4 ± 2.87
Stage W (%)	12.5 ± 2.19	12.9 ± 1.96	7.0 ± 1.37
Stage N1 (%)	11.6 ± 1.81	15.2 ± 2.32	8.7 ± 1.47
Stage N2 (%)	43.0 ± 4.20	42.5 ± 3.11	39.6 ± 2.96
Stage N3 (%)	15.4 ± 4.43	17.2 ± 3.10	25.4 ± 3.23
REM sleep (%)	17.4 ± 2.43	11.8 ± 2.25	19.5 ± 3.50
REM latency (min)	59.3 ± 7.94	63.4 ± 3.17	62.1 ± 3.82
SOL (min)	11.6 ± 1.53	8.9 ± 1.20	5.2 ± 1.02
WASO (min)	3.8 ± 1.13	5.4 ± 1.49	2.4 ± 0.68
SE (%)	87.5 ± 2.19	87.1 ± 1.96	93.0 ± 1.37
TIB (min)	84.8 ± 2.55	79.6 ± 2.69	80.1 ± 3.59

*Sleep parameters were obtained from the first sleep cycle because not all subjects had a second sleep cycle. REM latency, latency to REM sleep onset; SOL, sleep-onset latency; WASO, wake time after sleep onset; SE, sleep efficiency. TIB, time in bed, which indicates the duration of each sleep session (the time interval between lights-off and lights-on).*

### Anatomical MRI Acquisition and Region of Interest

Anatomical MRI data in Experiment 1 were acquired and used to determine the conductor geometry for the boundary element model (BEM) of the head (Hamalainen and Sarvas, 1989) and to register the EEG sensor locations with the individual subject’s anatomy (Dale et al., 1999; Fischl et al., 1999). Subjects were scanned in a 3T MR scanner (Trio, Siemens) using a 32-ch head coil. T1-weighted MR images (MPRAGE; TR = 2.531 s, TE = 3.28 ms, flip angle = 7°, TI = 1100 ms, 256 slices, voxel size = 1.3 mm × 1.3 mm × 1.0 mm) were acquired. The cortical surface was inflated for each subject for brain parcelation to localize individual gyri and sulci (Fischl et al., 2004).

Regions within the DMN were individually anatomically determined *a priori* based on previously published papers using an automated parcelation method (Fischl et al., 2004; Destrieux et al., 2010; Tamaki et al., 2016). We defined the DMN as a circuit that included the medial prefrontal, inferior parietal, and posterior parietal cortices, according to previous research (Mason et al., 2007; Raichle and Snyder, 2007; Tamaki et al., 2016). The medial prefrontal cortex consists of the anterior part of the superior frontal gyrus and the anterior cingulate gyrus and sulcus. The inferior parietal cortex consists of the inferior parietal gyrus and angular gyrus. The posterior parietal cortex consists of the precuneus gyrus, posterior-dorsal cingulate gyrus, and sup-parietal sulcus.

### Source Localization of EEG

To compute the strength of brain activities during sleep in the DMN, EEG data were subjected to the Morlet wavelet analysis in Experiment 1 and source localization using the minimum-norm estimate (MNE) of individual MRI information (see section “Anatomical MRI Acquisition and Region of Interest”). The Morlet wavelet analysis was applied to raw EEG data (Lin et al., 2004; Ahveninen et al., 2007; Tamaki et al., 2013) every 3 s to obtain the MNE strength at the peak frequency of 6 Hz (theta activity) and 1–4 Hz (1 Hz bin, delta activity) during REM sleep and every 30 s to obtain the MNE strength at 1–4 Hz (delta activity) during NREM sleep. The window width in the Morlet wavelet analysis was specified as 10 such that the MNE strength at 6 Hz would cover activities from 5 to 7 Hz, which corresponds to the theta band, and the strength at 1–4 Hz should cover activities from 1 to 4 Hz in the delta band. EEG during REM sleep was measured at a better temporal resolution (3 s) than that during NREM sleep because we classified REM sleep into tonic and phasic periods (see section “Classification of Phasic and Tonic Periods” below). To localize the current sources underlying the EEG signals, the cortically constrained MNE was used on EEGs using individual anatomical MRIs and constrained the current locations to the cortical mantle (Lin et al., 2004; Ahveninen et al., 2007). Information from the EEG sensor locations and structural MRI segmentation were used to compute the forward solutions for all source locations using a three-layer model of the boundary element method (BEM) (Hamalainen and Sarvas, 1989). The individual forward solutions constituted the rows of the gain (lead-field) matrix. The noise covariance matrix was computed from raw EEG data for 30 s during wakefulness. These two matrices were used to calculate the inverse operator to yield the estimated source activity during sleep, as a function of time, on a cortical surface (Lin et al., 2004; Ahveninen et al., 2007). The theta and delta-band activities were then averaged for tonic and phasic periods during REM sleep (see section “Classification of Phasic and Tonic Periods” below).

### Classification of Phasic and Tonic Periods

To classify EEG recordings during REM sleep into the phasic and tonic periods, we first detected eye movements during REM sleep automatically. First, a bandpass filter (0.5–8 Hz) was applied to the vertical and horizontal EOG electrodes during REM sleep. We then measured the amplitudes of eye movements. If the amplitude was 20 μV or higher, then it was counted as 1 movement. Eye movements that occurred within a brief time window (within 100 ms) were counted as only 1 movement. Based on the eye movements, the recordings during REM sleep were classified into a phasic or tonic period every 3 s according to previous studies (Takahara et al., 2002, 2006b). If at least one eye movement was detected within a 3-s epoch, the period was classified as a phasic period, and if no eye movements were detected within a 3-s epoch, the period was classified as a tonic period. We measured the percentage of phasic periods in REM sleep (Supplementary Table 1). We also measured the REM density by measuring the number of REMs divided by (1) the total duration of REM sleep (min) and (2) the duration of the phasic periods (min) that occurred during REM sleep. We compared these two types of REM density measures on day 1 in Experiments 1 vs. 2.

### Auditory Stimuli

Auditory stimuli were controlled using MATLAB (The MathWorks, Inc.) software and were presented through earphones (HAFR6A, JVC Americas, Corp.). The stimuli consisted of 2000-Hz deviant (presented at 10% probability) and 1000-Hz standard (presented at 90% probability) pure tones, all of which were 50 ms in duration (10 ms rise/fall). These sounds were presented monaurally every 1 s (1 trial = 1000 ms, fixed ISI = 950 ms). The probabilities of the sound type (deviant or standard) and the presented ear (left or right) were pseudorandomized every 30 s, which corresponded to the sleep-stage scoring epoch (see section “Sleep-Stage Scoring and Sleep Parameters”). More concretely, 30 sounds were presented in total, 15 per ear, in a given 30-s epoch. The probability of a deviant sound occurring was 10%, and at least one deviant sound was presented to each ear.

The sound intensity was approximately 35 dB (Extech 407740, Digital Sound Level Meter, Extech Instruments, Corp.), which was lower than that presented in previous studies that also used an oddball paradigm during REM sleep (50–100 dB) (Sallinen et al., 1996; Cote and Campbell, 1999a; Cote et al., 1999, 2001; Takahara et al., 2002, 2006b) to prevent subjects from waking. It was confirmed that 35 dB was sufficiently quiet to maintain sleep in each subject before the sleep session began.

### Analysis of Brain Responses to Auditory Stimuli

We examined the evoked brain potential known as P2 (Takahara et al., 2002, 2006b) from EEG data recorded during REM sleep using the oddball paradigm in Experiment 2. P2 is a positive brain potential that appears during REM sleep, and its amplitude increases to rare and salient stimuli (Takahara et al., 2002, 2006b). Therefore, P2 responses are used as an index of vigilance during REM sleep in humans (Takahara et al., 2006b).

To obtain the P2 amplitudes, first, six channels (three channels per hemisphere) from the central site (left: C1, C3, and C5; right: C2, C4, and C6) were analyzed. This site was chosen because it had the largest amplitudes in the previous study (Takahara et al., 2006b). Second, we also measured the P2 amplitudes from fronto-central electrodes (left: FC1, FC3, and FC5; right: FC2, FC4, and FC6) because this site also showed higher amplitudes in the topographic maps (see Figure 3). All data were examined visually for each trial, and any trials that included arousal (Bonnet et al., 1992; Iber et al., 2007) or motion artifacts were excluded from further analyses.

Analyses of brain responses followed a previous study (Tamaki et al., 2016). The EEG amplitudes during the 200-ms prestimulus period (−200 to 0 ms) were averaged. The mean EEG amplitude from the prestimulus period was subtracted from the EEG amplitudes from the 0- to 1000-ms post-stimulus period to apply baseline correction to the signal amplitude for each of the 1-s trials (0 to 1000-ms post-stimulus). These values were averaged for each sound type (deviant and standard), hemisphere (left and right), day (day 1 and day 2), and period (tonic and phasic) during REM sleep to compute averaged brain responses. The maximum value (peak) of the 150–250 ms post-stimulus time window from the brain responses was used as the P2 amplitude. We chose this time window because it roughly corresponded to the P2 window defined in previous studies (Bastuji et al., 1995; Perrin et al., 1999; Crowley and Colrain, 2004; Takahara et al., 2006b). Averaged values were obtained for each of the phasic and tonic periods (see section “Classification of Phasic and Tonic Periods”). For visualization purposes (Figure 2 and Supplementary Figure 2), a moving average with a 50-ms window (2 ms step size) was applied to amplitude values to smooth the waveforms. The area under the curve (Figure 5 and Supplementary Figure 7) was measured by summing all the positive values in a 150–250 ms window for the deviant trials for each hemisphere, period, and day.

Topographic maps (Figure 3 and Supplementary Figures 3, 4) were made for the P2 amplitudes across electrode locations. For each electrode, the peak in the 150–250 ms post-stimulus time window was averaged across subjects. To control for interindividual differences in amplitudes across electrode locations, the values were z-transformed for each subject across all the electrode locations. Z-transformation was applied only for the data used to make the topographic maps.

### Statistical Analyses

An α level (type-I error rate) of 0.05 was set for all statistical analyses. In Experiment 1, a two-tailed paired *t*-test was performed for analyses of delta activity during stage N3 sleep in the left vs. right hemispheres. Three-way repeated measures ANOVA on the measured delta and theta strengths during REM sleep (factors: frequency, period, hemisphere) was performed. In Experiment 2, a paired *t*-test was performed on sleep parameters. Three-way repeated measures ANOVA was used on each of the P2 amplitudes and the area under the curve. For *post hoc* analysis, two-tailed *t*-tests were used with Bonferroni correction as a multiple comparisons test. When the Bonferroni correction was used, the p values shown were adjusted values multiplied by the number of comparisons. When a *t*-test indicated a statistically significant difference, the effect size was calculated using Cohen’s *d* (Cohen, 1988), which indicates the magnitude of the difference between the two groups. This result is interpreted as having a large effect size when Cohen’s *d* ≥ 0.8 and as having a medium effect size when *d* ≥ 0.5.

## Results

### Confirmation of the FNE

**FIGURE 1:**
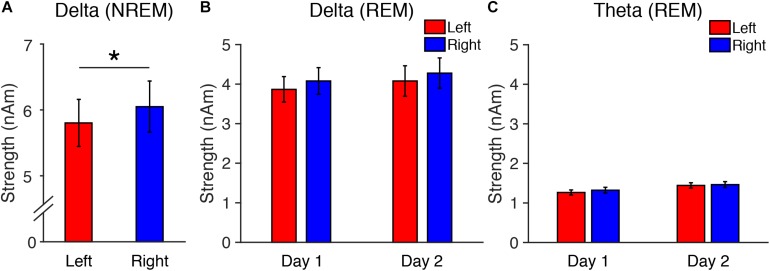
Source-localized oscillatory activity in the DMN in Experiment 1. **(A)** Delta strength during NREM sleep on Day 1, and delta **(B)** and theta **(C)** strength during REM sleep. *N* = 12. ^∗^*p* < 0.05.

In Experiment 1, we compared the sleep-onset latency between day 1 and day 2. We found that the sleep-onset latency, which is a critical measure of the FNE (Schmidt and Kaelbling, 1971; Webb and Campbell, 1979; Tamaki et al., 2005a), was significantly longer on day 1 (11.6 ± 1.53 min) than on day 2 (7.3 ± 1.56 min) [paired *t*-test, *t*(11) = 2.42, *p* = 0.034, Cohen’s *d* = 0.70], confirming the FNE in Experiment 1. In addition, we found an interhemispheric asymmetry in delta activity during NREM sleep, a signature of the FNE, in Experiment 1 (see below, Figure 1A).

To confirm that the FNE occurred in Experiment 2, we compared sleep-onset latency between day 1 and day 2. We found that the sleep-onset latency was significantly longer on day 1 than day 2 [paired *t*-test, *t*(11) = 5.01, *p* < 0.001, Cohen’s *d* = 1.47]. Thus, we confirmed that the FNE had occurred in Experiment 2 (Table 1).

Additionally, in Experiment 2, the WASO was significantly larger [paired *t*-test, *t*(11) = 2.74, *p* = 0.019, Cohen’s *d* = 0.79, uncorrected for multiple comparisons], and the percentage of time spent in stage N3 sleep was significantly lower [paired *t*-test, *t*(11) = 2.20, *p* = 0.049, Cohen’s *d* = 0.64, uncorrected for multiple comparisons] on day 1 than day 2. These results indicate that it took longer to fall asleep on day 1 than on day 2, sleep was more fragmented, and deep sleep was reduced, in accordance with previous studies (Kales et al., 1967a; Scharf et al., 1975; Webb and Campbell, 1979; Tamaki et al., 2016). However, there was no significant difference in the duration of [paired *t*-test, *t*(11) = 1.15, *p* = 0.151] or latency to [paired *t*-test, *t*(17) = 0.27, *p* = 0.794] REM sleep. Although we did not compare between days in Experiment 1 statistically, the sleep parameters on day 1 in Experiment 1 were similar to those on day 1 in Experiment 2 (e.g., sleep-onset latency, stage N3%). See Table 1 for the results of all the sleep parameters for Experiments 1 and 2.

### Experiment 1

#### Source-Localized Delta and Theta Activity

Experiment 1 examined whether there was interhemispheric asymmetry in delta or theta activity in the DMN during REM sleep in association with the FNE. This experiment also examined whether interhemispheric asymmetry in delta activity was present during stage N3 of NREM sleep, as in the previous study (Tamaki et al., 2016). We measured delta (1–4 Hz) and theta activity (∼5–7 Hz) originating in the DMN in each hemisphere during REM sleep and measured delta activity (1–4 Hz) during stage N3 sleep (see section “Source Localization of EEG”).

First, we performed a two-tailed paired *t*-test on the measured delta strength during stage N3 between the left and right hemispheres. Because one subject did not show stage N3 sleep, the analysis of delta strength included data from only 11 subjects. We confirmed that interhemispheric asymmetry was present in delta activity in the DMN during stage N3 of NREM sleep [*t*(10) = 2.34, *p* = 0.042, Cohen’s *d* = 0.70]. Delta activity was significantly lower in the left than the right hemisphere (Figure 1A). This result replicates previous findings (Tamaki et al., 2016).

Second, we performed three-way repeated measures ANOVA on the delta and theta strengths during REM sleep (factors: Frequency, Period, Hemisphere, see Figures 1B,C). The ANOVA results indicated that the main effects of Period [*F*(1,11) = 20.22, *p* = 0.001] and Frequency [*F*(1,11) = 63.14, *p* < 0.001] were significant. Other factors or interactions between factors were not significant [a main effect of Hemisphere: *F*(1,11) = 2.61, *p* = 0.134; Frequency × Period: *F*(1,11) = 0.78, *p* = 0.396; Frequency × Hemisphere: *F*(1,11) = 1.07, *p* = 0.324; Period × Hemisphere: *F*(1,11) = 1.44, *p* = 0.256; Frequency × Period × Hemisphere: *F*(1,11) = 0.142, *p* = 0.713; Figures 1B,C]. These results demonstrate that delta and theta activity during REM sleep did not show interhemispheric asymmetry, while delta activity during stage N3 NREM sleep replicated previous findings showing interhemispheric asymmetry.

### Experiment 2

#### Evoked Brain Responses

We measured the P2 amplitude, which is one of the primary evoked components seen during REM sleep (Takahara et al., 2002, 2006b). The amplitude of P2 may differ based on the eye movement state. Thus, we analyzed brain responses during the tonic (no REMs) and phasic (1 or more REMs) periods of REM sleep (see section “Classification of Phasic and Tonic Periods” and Supplementary Table 1 for the percentage of time spent in the phasic period). To test whether the brain was more vigilant in one hemisphere during REM sleep with the FNE and whether the level of vigilance differed based on the eye movement state, we measured the mean amplitudes of P2 for each hemisphere (left vs. right), period (phasic vs. tonic), and sound type (deviant vs. standard) (see section “Analysis of Brain Responses to Auditory Stimuli” for measurement of amplitude). Statistical tests were performed using six subjects whose data were available for both day 1 and day 2 for within-subjects comparisons. However, the figures are shown in the following two ways: (1) data using the six subjects and (2) using all the available data treated independently in Supplementary Figures (*n* = 8 for day 1, and *n* = 10 for day 2).

**FIGURE 2:**
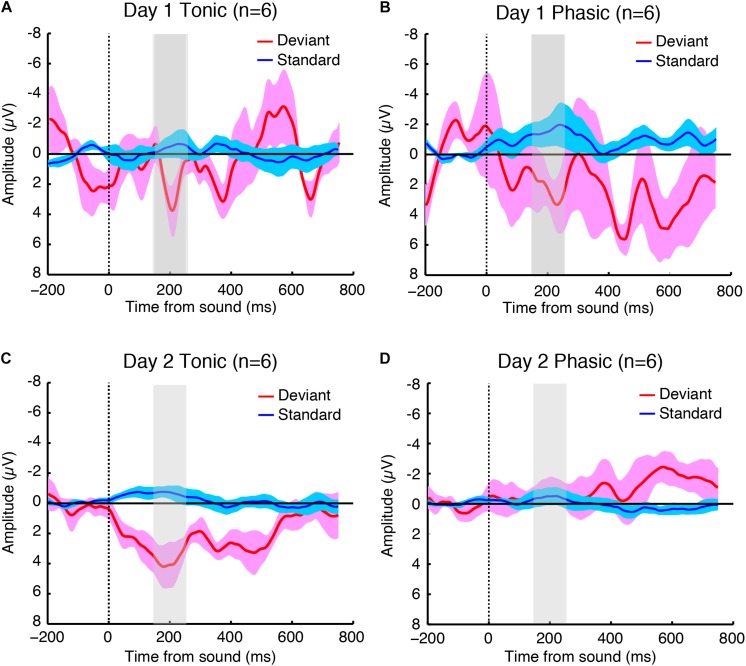
Grand-averaged brain responses of six subjects (averaged across both hemispheres because the statistical tests indicated no significant difference between hemispheres) to deviant (red) and standard (blue) tones time-locked to sound onset during the **(A)** tonic and **(B)** phasic periods on day 1, and the **(C)** tonic and **(D)** phasic periods on day 2 during REM sleep. The shaded part indicates 150–250 ms window where the P2 amplitudes were measured. *N* = 6.

**FIGURE 3:**
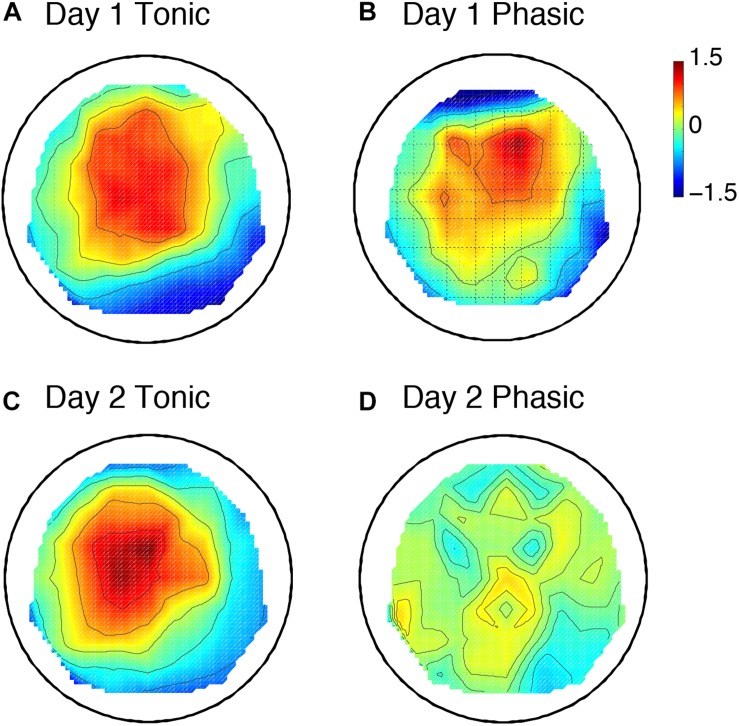
Topographic maps including 6 subjects’ data for the P2 amplitude. Day 1 tonic **(A)** and phasic **(B**), and day 2 tonic **(C)** and phasic **(D**). *N* = 6. The values are z-transformed (see section “Materials and Methods”).

The sleep session on day 2 was treated as a normal sleep without the FNE. On day 2, a clear P2 was elicited to deviant tones during the tonic period (Figure 2C and Supplementary Figure 2C), and no visible P2 was elicited during the phasic period (Figure 2D and Supplementary Figure 2D) in the ground-averaged brain responses and topographic maps (Figures 3C,D and Supplementary Figures 3C,D). However, on day 1, with the FNE, a large P2 was elicited to deviant tones during both tonic and phasic periods [Figures 2A,B and Supplementary Figures 2A,B in the ground-averaged brain responses, and Figures 3A,B and Supplementary Figures 3A,B for the topographic maps, Supplementary Figure 4 for the topographic maps that indicate day difference (day 1 minus day 2)].

**FIGURE 4:**
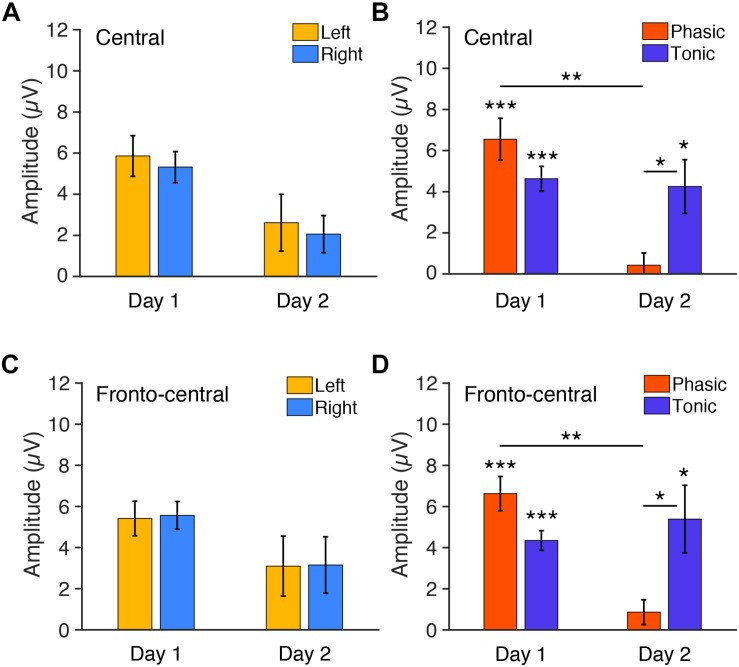
Mean P2 amplitudes to deviant tones during REM sleep measured from **(A,B)** central and **(C,D)** fronto-central electrodes. **(A)** The amplitudes for each hemisphere on days 1 and 2 (phasic and tonic periods averaged). *N* = 6. **(B)** The amplitudes for each phasic and tonic period on days 1 and 2 (hemispheres averaged). *N* = 6. *Post hoc* paired *t*-test, ^∗∗∗^*p* < 0.001, ^∗∗^*p* < 0.01, ^∗^*p* < 0.05 (Bonferroni correction). **(C)** The amplitudes for each hemisphere on days 1 and 2 (phasic and tonic periods averaged). *N* = 6. **(D)** The amplitudes for each phasic and tonic period on days 1 and 2 (hemispheres averaged). *N* = 6. *Post hoc* paired *t*-test, ^∗∗∗^*p* < 0.001, ^∗∗^*p* < 0.01, ^∗^*p* < 0.05 (Bonferroni correction).

We next tested whether the FNE impacts P2 amplitudes. For the statistical tests below, we used six subjects’ data, as their data were available for both days 1 and 2. We first examined the P2 amplitudes measured from the central electrodes. Three-way repeated measures ANOVA was conducted with the within-subjects factors of Period (tonic, phasic), Hemisphere (left, right), and Day (day 1 vs. day 2). If there was interhemispheric asymmetry in the P2 amplitudes associated with the FNE, then a significant Day × Hemisphere interaction should be present. If the amplitudes in a period were different between days, then a significant Day × Period interaction should be present. The statistical results showed that the Day × Hemisphere interaction was not significant [Figure 4A, *F*(1,5) < 0.01, *p* = 0.99]. However, there was a significant Period × Day interaction [Figure 4B, *F*(1,5) = 7.43, *p* = 0.042]. None of the main factors or other interactions [Hemisphere: *F*(1,5) = 0.62, *p* = 0.466; Period: *F*(1,5) = 1.06, *p* = 0.350; Day: *F*(1,5) = 3.70, *p* = 0.112; Hemisphere × Period: *F*(1,5) = 0.64, *p* = 0.462; Day × Hemisphere × Period: *F*(1,5) = 0.88, *p* = 0.390] were significant.

Because the Period × Day interaction was significant, we performed *post hoc* analyses to investigate the source of this interaction. Because no significant effect of Hemisphere was found in the above ANOVA, data from the left and the right hemispheres were pooled for subsequent analyses. *Post hoc t*-tests indicated a significant difference in the P2 amplitude between day 1 and day 2 in the phasic period [orange bars in Figure 4B; paired *t*-test, *t*(11) = 4.05, *p* = 0.008, Cohen’s *d* = 1.17; Bonferroni correction for four comparisons] and between the phasic and tonic periods on day 2 [Figure 4B; paired *t*-test, *t*(11) = 3.12, *p* = 0.039, Cohen’s *d* = 0.90, Bonferroni correction for four comparisons]. There was no significant difference between days in the tonic period [blue bars, Figure 4B; paired *t*-test, *t*(11) = 0.27, *p* = 1.00, Bonferroni correction for the four comparisons] or between the tonic and phasic periods on day 1 [Figure 4B; paired *t*-test, *t*(11) = 0.27, *p* = 1.00; Bonferroni correction for four comparisons]. We performed one-sample *t*-tests on the P2 amplitudes for each of the periods and days to investigate whether P2 was elicited, i.e., whether P2 amplitude was significantly different from zero (Figure 4B). The P2 amplitude was significantly different from 0 during the tonic period on day 1 [*t*(11) = 7.70, *p* < 0.001, Cohen’s *d* = 2.22, Bonferroni correction for the following four comparisons] and day 2 [*t*(11) = 3.25, *p* = 0.031, Cohen’s *d* = 0.94, Bonferroni correction for four comparisons] and was significantly different from zero during the phasic period on day 1 [*t*(11) = 6.43, *p* < 0.001, Cohen’s *d* = 1.86, Bonferroni correction for four comparisons] but not on day 2 [*t*(11) = 0.71, *p* = 1.00, Bonferroni correction for four comparisons]. Thus, the P2 amplitude was larger on day 1 than day 2 during the phasic period. See Supplementary Figures 5A,B for the results using all the available data (*n* = 8 on day 1, *n* = 10 on day 2).

In addition, we next examined the P2 amplitudes measured from the fronto-central electrodes, as the topographic maps suggest that this site shows higher amplitudes of P2. Three-way repeated measures ANOVA was performed on the P2 amplitudes from the fronto-central electrodes with the within-subjects factors of Period (tonic, phasic), Hemisphere (left, right), and Day (day 1 vs. day 2). If the amplitudes in a period were different between days, then a significant Day × Period interaction should be present. There was indeed a significant Period × Day interaction [*F*(1,5) = 9.08, *p* = 0.030]. The Day × Hemisphere interaction was not significant [Figure 4C; *F*(1,5) = 0.01, *p* = 0.938]. None of the main factors or other interactions [Hemisphere: *F*(1,5) = 0.04, *p* = 0.848; Period: *F*(1,5) = 0.96, *p* = 0.372; Day: *F*(1,5) = 2.22, *p* = 0.848; Hemisphere × Period: *F*(1,5) = 0.35, *p* = 0.580; Day × Hemisphere × Period: *F*(1,5) = 0.42, *p* = 0.548] were significant.

Because the Period × Day interaction was significant, we performed *post hoc* analyses to investigate the source of this interaction. Because no significant effect of Hemisphere was found in the above ANOVA, data from the left and the right hemispheres were pooled for subsequent analyses. *Post hoc t*-tests indicated a significant difference in the amplitude between day 1 vs. day 2 in the phasic period [Figure 4D; paired *t*-test, *t*(11) = 4.60, *p* = 0.003, Cohen’s *d* = 1.46; Bonferroni correction for the following four comparisons] and between the phasic and tonic periods on day 2 [Figure 4D; paired *t*-test, *t*(11) = 2.98, *p* = 0.0496, Cohen’s *d* = 0.83, Bonferroni correction for four comparisons]. There was no significant difference between days in the tonic period [Figure 4D; paired *t*-test, *t*(11) = 0.64, *p* = 1.00, Bonferroni correction for the four comparisons] or between the tonic and phasic periods on day 1 [Figure 4D; paired *t*-test, *t*(11) = 2.76, *p* = 0.072; Bonferroni correction for four comparisons]. We performed one-sample *t*-tests on the amplitudes for each of the periods and days to investigate whether P2 was elicited, i.e., whether P2 amplitude was significantly different from zero (Figure 4D). The amplitude was significantly different from 0 during the tonic period on day 1 [*t*(11) = 9.11, *p* < 0.001, Cohen’s *d* = 2.63, Bonferroni correction for four comparisons] and day 2 [*t*(11) = 3.27, *p* = 0.030, Cohen’s *d* = 0.94, Bonferroni correction for four comparisons], and was significantly different from zero during the phasic period on day 1 [*t*(11) = 7.97, *p* < 0.001, Cohen’s *d* = 2.30, Bonferroni correction for four comparisons], but not on day 2 [*t*(11) = 1.43, *p* = 0.722, Bonferroni correction for four comparisons]. Thus, the P2 amplitude was larger on day 1 than day 2 during the phasic period also in the fronto-central electrodes. See Supplementary Figures 5C,D for the results of the fronto-central P2 using all the available data (*n* = 8 on day 1, *n* = 10 on day 2). Together, these results demonstrate that vigilance during REM sleep, especially during the phasic period, was enhanced on day 1 with the FNE, but there was no interhemispheric asymmetry.

We conducted additional analyses to confirm that these P2 results were due to the FNE, but not due to noises. First, we tested whether the increased P2 amplitudes to deviant tones during the phasic period on day 1 were due to noises caused by smaller number of trials for deviant tones compared to those for the standard tones. We used the same number of trials (randomly selected) for the standard tones as for the deviant tones and measured the amplitude of P2 to the standard tones. We did not find any evidence that indicates that the P2 amplitude is elicited by smaller number of trials for standard tones (see Supplementary Figure 6). We conducted three-way ANOVA (factors: day, period, hemisphere) on the P2 amplitudes elicited to the standard tones with the smaller number of trials. Neither a significant main effect nor an interaction was found [Period: *F*(1,5) = 0.14, *p* = 0.727; Day: *F*(1,5) < 0.01, *p* = 0.986; Hemisphere: *F*(1,5) = 0.21, *p* = 0.668; Period × Day: *F*(1,5) = 0.30, *p* = 0.610; Period × Hemisphere: *F*(1,5) = 1.09, *p* = 0.344; Day × Hemisphere: *F*(1,5) = 4.34, *p* = 0.092; Period × Hemisphere × Day: *F*(1,5) = 0.94, *p* = 0.378]. Thus, we believe that P2 elicited to deviant tones during sleep is not due to noise induced by using a smaller number of trials. This result is consistent with previous studies showing that ERPs elicited by standard tones in the oddball paradigm are very small during sleep (Takahara et al., 2002; Tamaki et al., 2016) and during wakefulness (Tamaki et al., 2016). It has been shown that ERP amplitude is larger to infrequent events and very small to frequent events (Squires et al., 1975). Second, we investigated whether the significant amplitude increase to deviant tones on day 1 during the phasic period was due to fluctuations in brain responses during the prestimulus period. We measured the averaged amplitude during the prestimulus baseline period for each condition (phasic and tonic periods on day 1 and day2) measured from central electrodes. Then we tested whether the averaged amplitude was significantly different from zero using a one sample *t*-test. The results showed that the prestimulus baseline amplitudes were not significantly different from zero 0 in any of the conditions [one-sample *t*-test, hemisphere averaged, Tonic day 1, *t*(5) = 0.15, *p* = 0.890; Tonic day 2, *t*(5) = 0.64, *p* = 0.549; Phasic day 1, *t*(5) = 0.08, *p* = 0.936; Phasic day 2, *t*(5) = 0.41, *p* = 0.700]. These results indicate that the significant increase of P2 amplitudes on day 1 during the phasic period was not due to smaller number of trials used or the fluctuations in brain response during prestimulus baseline period.

**FIGURE 5:**
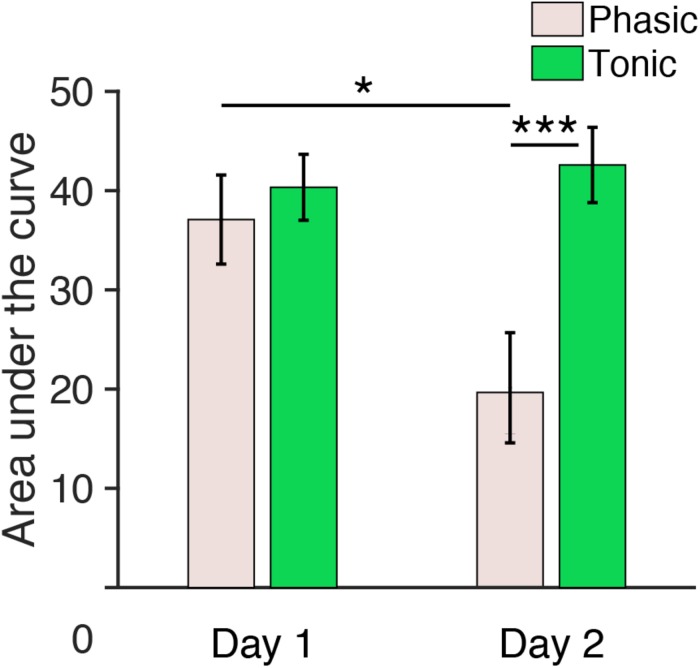
Area under the curve (150–250 ms) during REM sleep measured from central electrodes. The area under the curve for *n* = 6. *Post hoc* paired *t*-test, ^∗∗∗^*p* < 0.001, ^∗^*p* < 0.05 (Bonferroni correction).

We further measured the area under the curve of ERPs by summing all the positive values within a 150–250 ms window for each hemisphere, period, and day. Three-way repeated measures ANOVA with the within-subjects factors of Period (tonic, phasic), Hemisphere (left, right) and Day (day 1, day 2) was performed on the areas under the curve elicited by deviant tones (Figure 5). The statistical results were similar to the results for P2 amplitudes described above. There was a significant Period × Day interaction [*F*(1,5) = 8.80, *p* = 0.031]. The main effect of Period was also significant [*F*(1,5) = 7.48, *p* = 0.041]. None of the other main factors or interactions [Hemisphere: *F*(1,5) = 0.07, *p* = 0.801; Day: *F*(1,5) = 2.33, *p* = 0.188; Day × Hemisphere; *F*(1,5) = 0.03, *p* = 0.872; Hemisphere × Period: *F*(1,5) = 3.06, *p* = 0.141; Day × Hemisphere × Period: *F*(1,5) = 0.59, *p* = 0.478] were significant.

Because the Period × Day interaction was significant, we performed *post hoc* analyses to investigate the source of this interaction. Because no significant effect of Hemisphere was found in the above ANOVA, data from the left and the right hemispheres were pooled for subsequent analyses. *Post hoc t*-tests indicated a significant difference in the area under the curve between day 1 and day 2 during the phasic period [pale pink bars, Figure 5; paired *t*-test, *t*(11) = 3.01, *p* = 0.047, Cohen’s *d* = 0.87; Bonferroni correction for the following four comparisons] and between the phasic and tonic periods on day 2 [paired *t*-test, *t*(11) = 4.17, *p* < 0.001, Cohen’s *d* = 1.20; Bonferroni correction for four comparisons]. There was no significant difference in the area under the curve between days in the tonic period [green bars, Figure 5, paired *t*-test, *t*(11) = 0.90, *p* = 1.00, Bonferroni correction for four comparisons] or between the tonic and phasic periods on day 1 [paired *t*-test, *t*(11) = 1.05; Bonferroni correction for four comparisons]. See Supplementary Figure 7 for the area under the curve measured from all the subjects available. Together, these results demonstrate that the area under the curve for P2 during the phasic period was enhanced on day 1 with the FNE without interhemispheric asymmetry.

We next examined whether the FNE alters the latency to the P2 peak (Table 2). The latency to the P2 peak was measured for each hemisphere (left vs. right), period (phasic vs. tonic), and day (day 1 vs. day 2). Statistical tests were conducted using six subjects’ data (whose data are available for both days 1 and 2) as follows. Three-way repeated measures ANOVA was performed on the P2 latencies, with the within-subjects factors of Period (tonic, phasic), Hemisphere (left, right), and Day (day 1, day 2). None of the main effects or interactions were significant [Hemisphere: *F*(1,5) = 0.69, *p* = 0.446; Period: *F*(1,5) = 0.95, *p* = 0.375; Day: *F*(1,5) = 1.15, *p* = 0.333; Period × Day: *F*(1,5) = 0.48, *p* = 0.520; Day × Hemisphere: *F*(1,5) = 0.06, *p* = 0.816; Hemisphere × Period: *F*(1,5) = 2.36, *p* = 0.185; Day × Hemisphere × Period: *F*(1,5) = 0.36, *p* = 0.574]. See Supplementary Table 2 for the P2 latency measured from all the available subjects.

**TABLE 2:** P2 latency (Mean ± SEM, ms).

	**Tonic**				**Phasic**			
	**Day 1**		**Day 2**		**Day 1**		**Day 2**	
	**Left**	**Right**	**Left**	**Right**	**Left**	**Right**	**Left**	**Right**
Mean	209.8	201.5	210.2	191.8	204.3	227.8	170.2	201.6
SE	10.14	11.79	12.63	11.43	16.74	13.23	7.11	12.00

**N* = 6. See the main text for statistical results.*

#### REM Density

One may wonder whether sound presentations increased REM density (Table 3). We measured REM density in the following two ways: (1) the number of REMs divided by the total REM sleep duration (min) and (2) the number of REMs divided by the total duration of the combined phasic periods during REM sleep (min). Then, we compared REM densities on day 1 in Experiment 1 to those on day 1 in Experiment 2. We conducted an unpaired *t*-test on the REM densities. We did not find a significant difference between experiments [see Table 3; all REM sleep, *t*(18) = 0.16, *p* = 0.877; only the phasic period, *t*(18) = 1.94, *p* = 0.069].

**TABLE 3:** REM density on Day 1 (Mean ± SE, number per min).

	**Experiment 1 (*N* = 12)**	**Experiment 2 (*N* = 8)**
REM density (all REM sleep)	12.1 (2.36)	12.1 (3.32)
REM density (only phasic period)	24.0 (2.07)	31.0 (3.52)

*REM density for all REM sleep was measured by the number of rapid eye movements divided by the total REM sleep duration in minutes (the first row). The REM density was only measured during phasic REM sleep and was determined by the number of rapid eye movements divided by the duration of phasic REM sleep in minutes.*

## Discussion

The present study found that the level of vigilance during REM sleep was higher on day 1 than day 2, specifically during the phasic period. However, interhemispheric asymmetry in evoked brain responses was not found during REM sleep. Neither delta nor theta activity during REM sleep showed interhemispheric asymmetry, but we replicated previous findings showing interhemispheric differences in delta activity during NREM sleep.

Notably, although brain responses to auditory stimuli did not show interhemispheric asymmetry associated with the FNE during REM sleep, a larger response to rare stimuli was found, specifically during the phasic period. Because the amplitude of an evoked brain response to deviant stimuli during sleep correlates with the degree of vigilance (Nielsen-Bohlman et al., 1991; Michida et al., 2005), the present results suggest that the FNE augments vigilance during REM sleep, especially when REMs are observed. These results demonstrate that a surveillance system using both hemispheres exists during the phasic period of REM sleep.

We found that the amplitudes of brain responses were larger on day 1 than on day 2, specifically during the phasic period. Why did the phasic period, but not the tonic period, show augmented evoked brain responses in association with the FNE? The brain is more sensitive in the monitoring of external stimuli during the tonic period than in the phasic period during normal sleep without the FNE (Takahara et al., 2002). This suggests that there may be no additional capacity available for information processing or attention to external stimuli during the tonic period, as the attention capacity is likely to be limited (Kahneman, 1973; Marois and Ivanoff, 2005). If attentional resources are not available for external monitoring during the tonic period, when there is a need to increase vigilance even further, such as when sleeping in an unfamiliar environment, this increase would have to occur outside the tonic period, i.e., during the phasic period. This compensatory action might explain why vigilance was enhanced during the phasic period during REM sleep in association with the FNE.

There are several differences between the current ERP findings and those in previous studies. First, previous studies (Cote and Campbell, 1999a; Cote et al., 2001) indicated that P300 was elicited during REM sleep in an oddball paradigm. However, we do not think that the P300 component was elicited in the present study. The P300 component is known to be distributed over the posterior site, and its latency could be as early as 250 ms (Polich, 2007). However, the topographic maps did not indicate that the peak of the component in the present study was around the posterior site during the tonic or phasic periods. Second, previous studies (Sallinen et al., 1996; Takahara et al., 2006b) showed that a small P2 was elicited during both the tonic and phasic periods during REM sleep. However, P2 was not significantly elicited during the phasic period on day 2 in the present study. These inconsistencies could be due to the difference in the intensity of the sounds used across studies. It has been shown that the intensity of sounds affects ERP amplitudes during REM sleep (Cote and Campbell, 1999b). Indeed, previous studies have used much louder sounds (50–100 dB) (Sallinen et al., 1996; Cote and Campbell, 1999a; Cote et al., 1999, 2001; Takahara et al., 2002, 2006b) than those used in the present study (∼35 dB). In particular, these previous studies that showed P300 during REM sleep used very loud sounds (95∼100 dB). We speculate that loud sounds could increase vigilance during REM sleep and might even wake up subjects. This possible increase in vigilance might result in P300 during the phasic periods in the previous studies.

The phasic period of normal REM sleep without the FNE is linked to subjective mental activities (Berger and Oswald, 1962; Weinstein et al., 1988). Notably, a previous study suggested that sensitivity to external stimuli was lower during sleep-onset dreaming (Michida et al., 2005). Thus, the reason for a very small brain responses during the phasic period of normal REM sleep without the FNE may be due to ongoing mental activities, including dreaming, which may interfere with the monitoring of the external environments (Sallinen et al., 1996; Michida et al., 2005). However, a large brain response was elicited during the phasic period during REM sleep in association with the FNE. This result suggests that resources for internal mental activities are deployed for external monitoring when sleeping in an unfamiliar environment leading to the FNE.

We did not find clear interhemispheric asymmetry in delta or theta activity or in the amplitudes of the evoked potentials between hemispheres during REM sleep in association with the FNE. These results contrast our previous study (Tamaki et al., 2016), in which we found interhemispheric asymmetry in regional slow-wave activity and vigilance during deep NREM sleep. This difference suggests that different mechanisms are involved in surveillance during REM sleep and deep NREM sleep. It may be the case that since the arousal threshold may be too high and costly during deep NREM sleep to increase vigilance in both hemispheres, only one hemisphere is used for surveillance. However, resources for dreaming and mental activity are already available during REM sleep. These resources may be easily deployed for surveillance without much sacrifice in sleep. Thus, the surveillance is present in both hemispheres during REM sleep.

Some may wonder whether the circadian timing may affect the surveillance system associated with the FNE during REM sleep. A previous study (Tamaki et al., 2016) indicated that the FNE occurs regardless of whether a sleep period occurred during daytime nap or at night. Another previous study (Toussaint et al., 1997) did not show a significant effect of sleep cycle on the FNE during REM sleep (see Figure 1 in Toussaint et al., 1997). These studies suggest that the effect of the circadian rhythm on the FNE during REM sleep may not be large enough to alter the findings of the present study. However, how circadian phases influence the surveillance system with the FNE during REM sleep needs to be systematically clarified in future research.

The present study targeted the DMN for the analysis of brain activities during REM sleep, because a previous study (Tamaki et al., 2016) suggested that the DMN may be involved in the surveillance system in association with the FNE. However, whether the responsive brain network related to the FNE during REM sleep resides specifically in the DMN is still speculative. Other brain networks need to be investigated in future research.

## Conclusion

The protective surveillance system activated during REM sleep may involve a different mechanism than that activated during deep NREM sleep. A surveillance system was shown to increase vigilance in both hemispheres throughout REM sleep, specifically during the phasic period. This REM-specific surveillance system may be based on resources available for internal mental activity.

## Data Availability Statement

The datasets generated for this study are available on request to the corresponding author.

## Ethics Statement

The studies involving human participants were reviewed and approved by Institutional review board, Brown University. The patients/participants provided their written informed consent to participate in this study.

## Author Contributions

MT and YS designed the research and wrote the manuscript. MT performed the experiments and analyzed the data.

## Conflict of Interest

The authors declare that the research was conducted in the absence of any commercial or financial relationships that could be construed as a potential conflict of interest.
