# Supplementary material for: Surveillance During REM Sleep for the First-Night Effect
Source: Front Neurosci. 2019 Oct 29;13:1161. doi: 10.3389/fnins.2019.01161 (PMC6828645; doi:10.3389/fnins.2019.01161)
Supplement: Supplementary file 1 [file Data_Sheet_1.PDF]

Supplementary Table 1. The percentage of phasic periods during REM sleep (%, mean ± SE). The percentage of phasic period on day 1 was not significantly different between Experiment 1 and 2 ($t(18) = 0.90$, $p = 0.381$).

Exp.1	Exp.2	
Day 1 (N=12)	Day 1 (N=8)	Day 2 (N=10)
36.1 (6.38)	44.7 (6.81)	55.8 (5.27)

Supplementary Table 2. P2 latency measured from all the subjects. Day 1, n=8. Day 2, n=10.

	Tonic				Phasic			
	Day 1		Day 2		Day 1		Day 2	
	Left	Right	Left	Right	Left	Right	Left	Right
Mean	223.3	213.0	217.0	200.7	205.7	223.3	176.0	207.0
SE	6.01	11.87	15.59	12.38	20.36	17.64	10.46	16.07

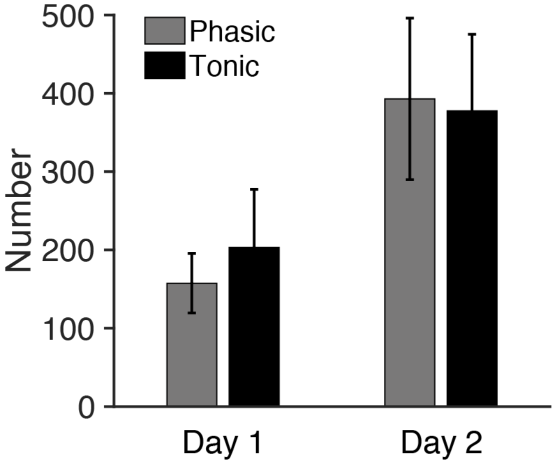

Supplementary Figure 1. The total number of sound presentations, including both the standard and deviant tones, in Experiment 2. A paired t-test was conducted on the number of sound presentations for each of days 1 and 2, separately, to test whether the number of sound presentations differed between the tonic vs. phasic periods. There was no significant difference in the number of sound presentation between the periods either on day 1 ($t(7) = 0.91$, $p = 0.394$) or day 2 ($t(9) = 0.59$, $p = 0.571$). N = 8 on day 1, n = 10 on day 2.

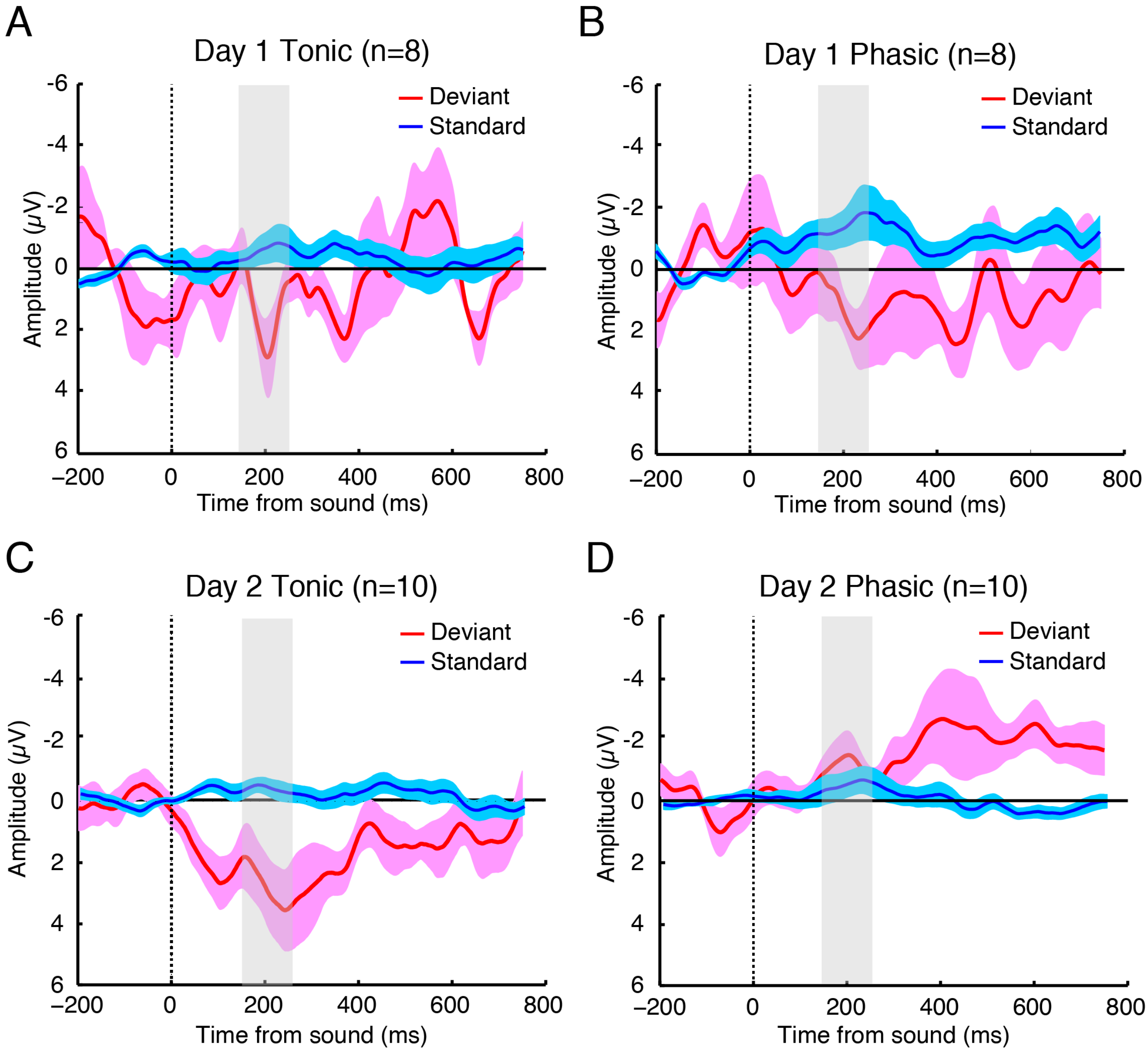

Supplementary Figure 2. The grand-averaged brain responses of all the data (averaged across both hemispheres because the statistical tests indicated no significant difference between hemispheres) to deviant (red) and standard (blue) tones time-locked to the sound onset during the (**A**) tonic and (**B**) phasic periods on day 1 (n = 8), and the (**C**) tonic and (**D**) phasic and tonic periods on day 2 (n = 10) during REM sleep. The shaded part indicates 150-250ms window where the P2 amplitudes were measured.

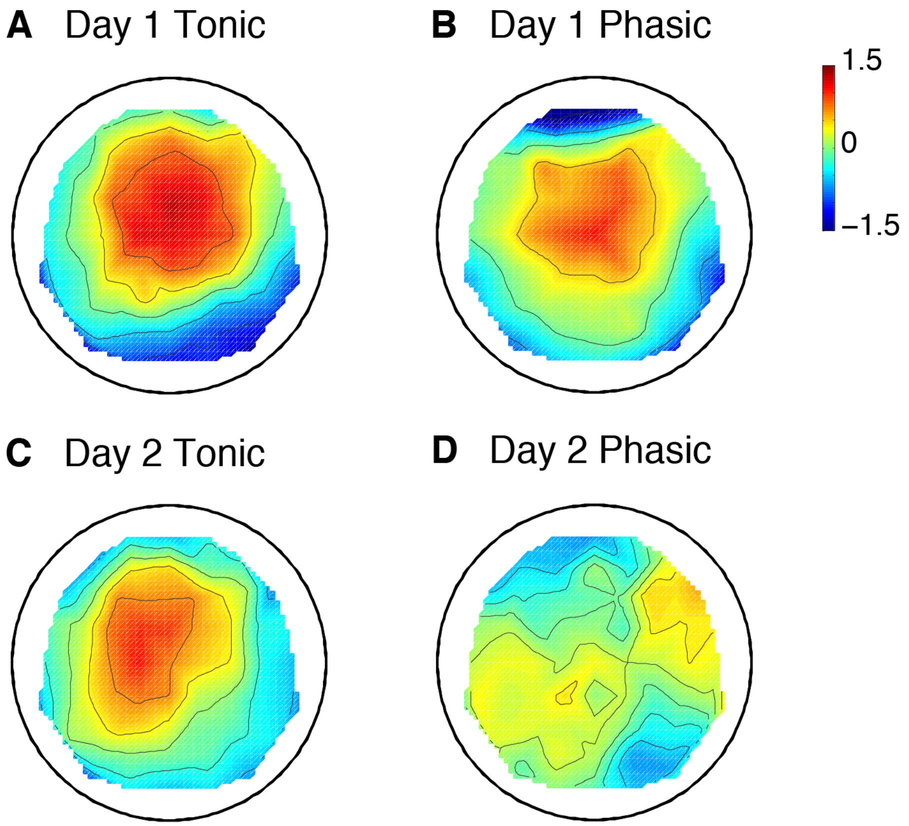

Supplementary Figure 3. Topographic maps including all subjects' data for the P2 amplitude. Day 1 tonic (**A**) and phasic (**B**), n = 8, and Day 2 tonic (**C**) and phasic (**D**), n = 10. The values are z-transformed (see **Materials and Methods**).

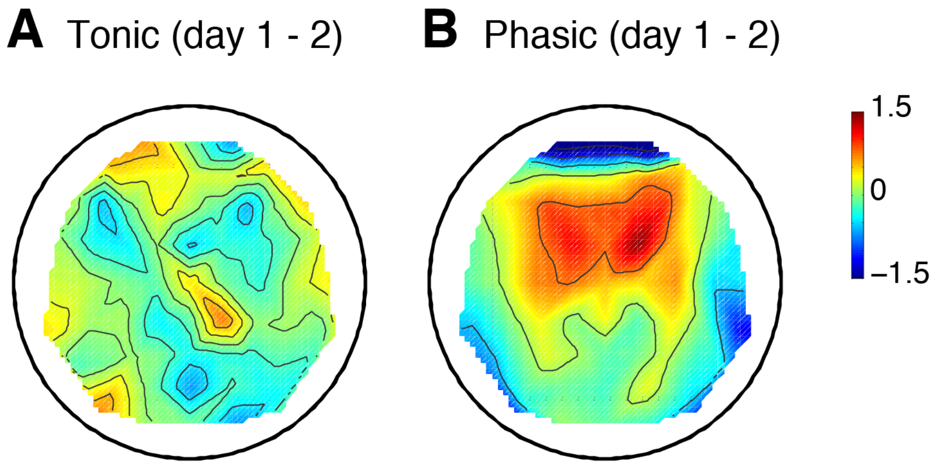

Supplementary Figure 4. Topographic maps for day 1 minus day 2 (day difference) for the P2 amplitude. Tonic (**A**) and phasic (**B**), including 6 subjects' data whose data were available for both days 1 and 2. The values are z-transformed (see **Materials and Methods**).

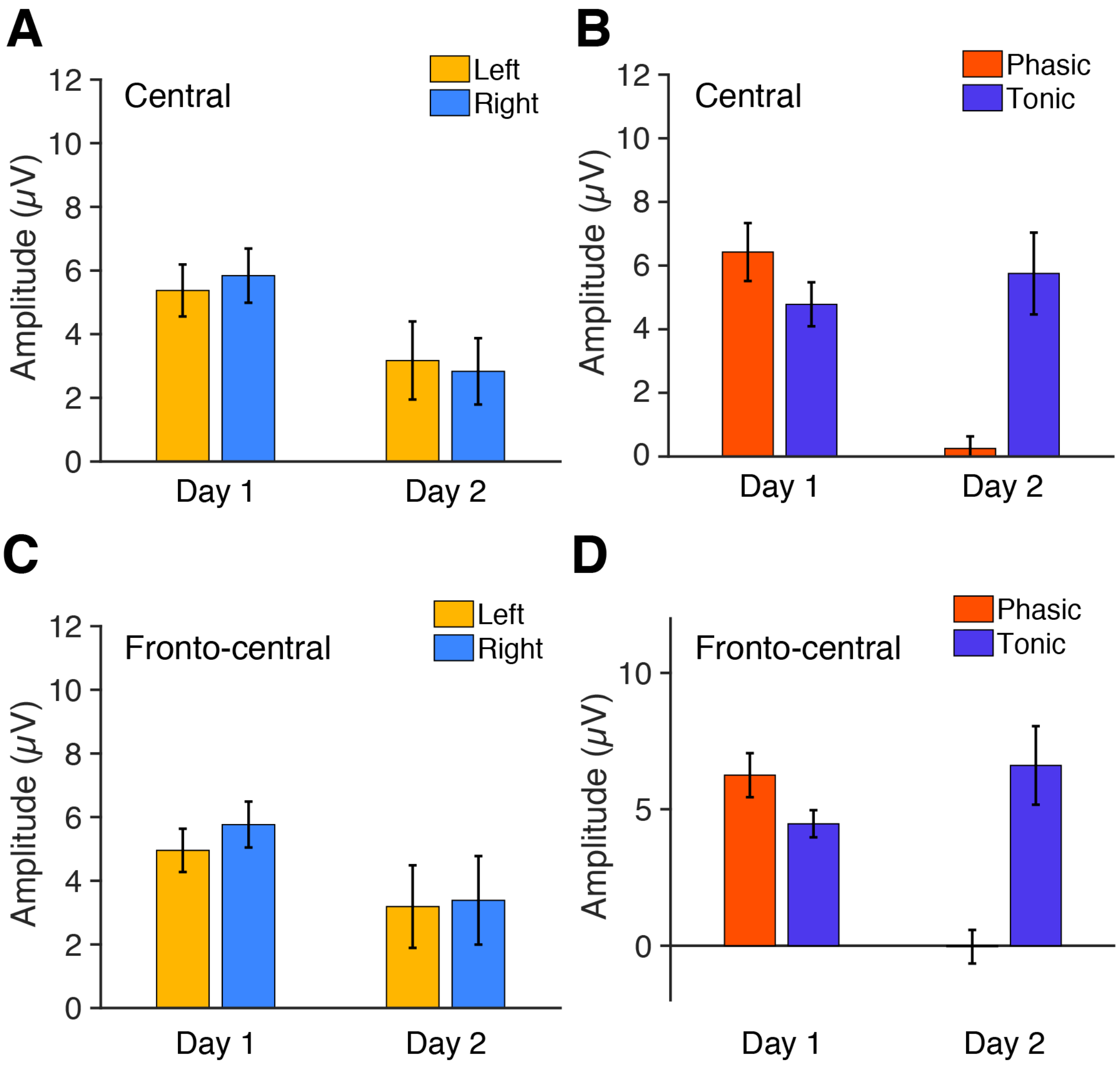

Supplementary Figure 5. Mean P2 amplitudes to deviant tones during REM sleep measured from (**A, B**) central and (**C, D**) fronto-central electrodes using all the available data. N = 8 for day 1 and n = 10 for day 2. (**A**) The amplitudes for each hemisphere on days 1 and 2 (phasic and tonic periods averaged). (**B**) The amplitudes for each phasic and tonic period on days 1 and 2 (hemispheres averaged). (**C**) The amplitudes for each hemisphere on days 1 and 2 (phasic and tonic periods averaged). (**D**) The amplitudes for each phasic and tonic period on days 1 and 2 (hemispheres averaged).

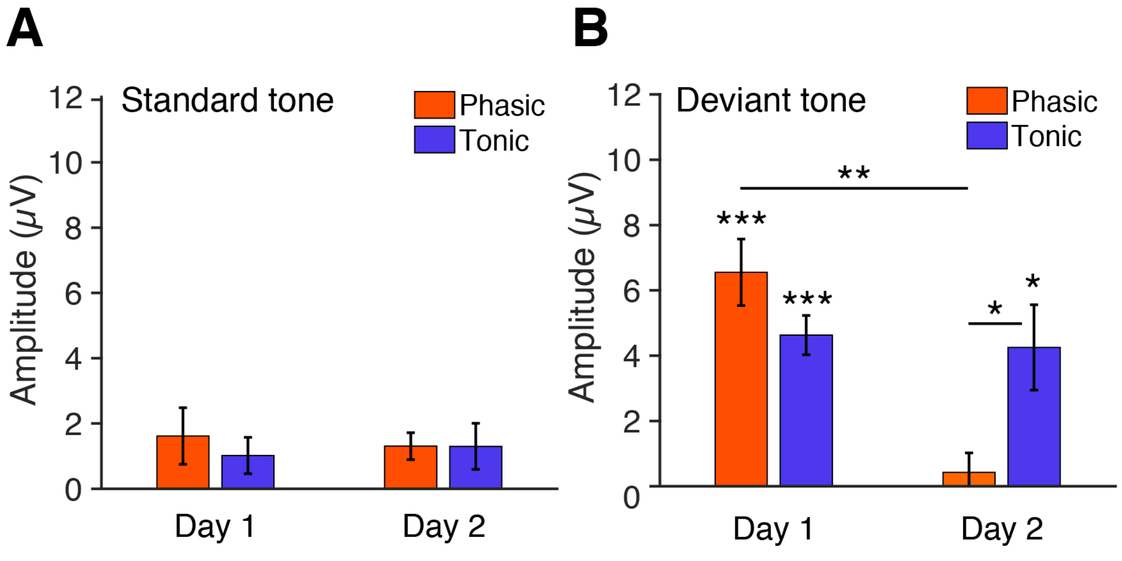

Supplementary Figure 6. The P2 amplitudes for the standard and deviant tones using the same number of trials. (**A**) The P2 amplitudes measured for the standard tones using the same number of trials as for the deviant tones. (**B**) The P2 amplitude for the deviant tones; the same figure as Figure 4B. See the main text for ANOVA results. N = 6 whose data were available for both days 1 and 2.***$p < 0.001$, **$p < 0.01$, *$p < 0.05$ (after Bonferroni correction).

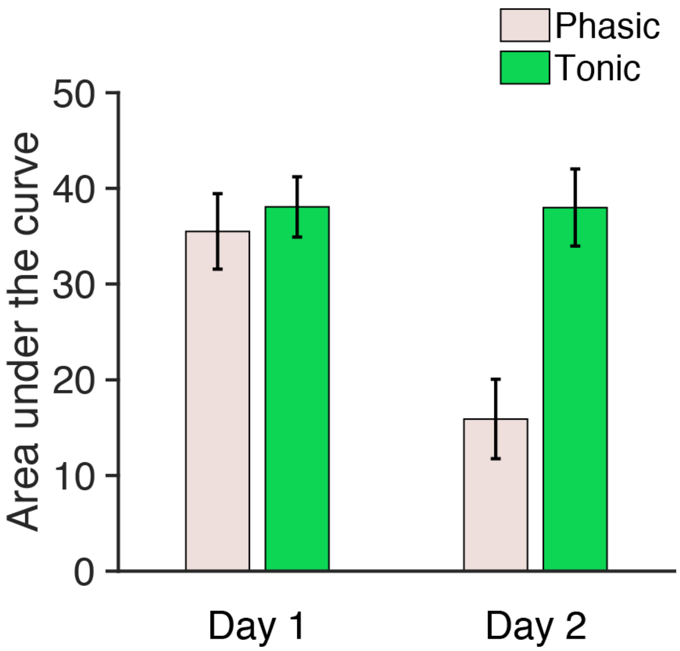

Supplementary Figure 7. Area under the curve (150-250 ms) during REM sleep measured from central electrodes. The area under the curve using all the available data (n = 8 for day 1, n = 10 for day 2).
